# How Convincing Is a Crowd? Quantifying the Persuasiveness of a Consensus for Different Individuals and Types of Claims

**DOI:** 10.1177/09567976251344549

**Published:** 2025-06-11

**Authors:** Manikya Alister, Keith Ransom, Saoirse Connor Desai, Ee Von Soh, Brett Hayes, Andrew Perfors

**Affiliations:** 1 University of Melbourne, School of Psychological Sciences; 2 University of Adelaide, School of Computer and Mathematical Sciences; 3 University of Technology Sydney, Graduate School of Health; 4 University of New South Wales, School of Psychology

**Keywords:** consensus, persuasion, source independence, social reasoning, individual differences

## Abstract

A powerful cue when reasoning is whether an apparent consensus has been reached. However, we do not yet know how the strength of this cue varies between different individuals and types of claims. In the current study (*N* = 78 U.S. adults, recruited from Prolific), we evaluated this with a realistic mock social-media paradigm in which each participant evaluated 60 diverse, real-world claims based on posts from people who either disagreed with each other, formed a consensus independently, or formed a consensus using shared sources. Almost all participants revised their beliefs to align with the consensus; many also qualitatively changed their minds. A consensus was also more persuasive for claims more likely to have a ground truth (i.e., more knowable claims). Although most people were insensitive to consensus independence, some were more persuaded by a consensus formed independently, whereas some were equally convinced by a consensus formed using the same sources.

When encountering a new opinion or claim, it is often difficult to quickly and accurately verify its truth on the basis of personal experience alone. We might approach this situation by relying on cues, such as the number of people who agree with it. For instance, if you come across a social-media post stating that “genetically modified crops are a good idea,” you might be inclined to give this claim more weight if there is a consensus supporting it.

This kind of *consensus effect*, in which people tend to be more convinced by something if multiple people agree on it, has been demonstrated extensively (see Mercier & Morin, 2019, and Oktar & Lombrozo, 2025, for reviews). However, reasoning about consensus in everyday life can be complex. For one thing, people vary generally in how much they engage in critical reasoning (Cacioppo & Petty, 1982; Kruglanski et al., 1993). Another issue is that many factors go into evaluating complex claims, and individuals differ in how they weigh those factors. These factors include the prestige of (Atkisson et al., 2012) or the confidence in (Sah et al., 2013) the source of the claim, and the complexity of the arguments given in support of it (Zemla et al., 2017). Moreover, reasoners realize that people making arguments in support of a claim (e.g., on social media) have both different levels of competence (Lin et al., 2016) and different goals, which include persuasion, identity signaling, trolling, and deception (Pucci et al., 2023). If individuals have different assumptions about the nature or importance of these factors, this will mean that they differ in how sensitive they are to consensus effects in the first place. Indeed, research suggests people differ in the extent to which they consider social information when making judgments and decisions (Molleman et al., 2019; Toelch et al., 2014). However, previous research has typically focused on group-level rather than individual behavior.

Another key factor is the independence between the sources that contribute to the consensus. It is often argued that a consensus should be more convincing when everyone within the consensus reached their opinions independently (Connor Desai et al., 2022; Harkins & Petty, 1987; Whalen et al., 2018; Xie & Hayes, 2022; Yousif et al., 2019). If multiple social-media posts from different people agree that genetically modified crops are a good idea, and they all reference different, independent sources supporting this claim (e.g., different scientific studies or surveys), one would think that this sort of *independent consensus* would be more convincing than if each of the people involved referenced the same source (*dependent consensus*). Indeed, normative models of decision-making support the idea that people should weigh claims corroborated by multiple independent sources more than claims corroborated by dependent (repeated) sources (for a review, see Couch, 2022).

Unfortunately, in the real world, it is often unclear which primary sources have influenced someone’s opinions. Even if the primary sources that influenced people are known, one might be unsure whether those sources are truly independent, because they could have collaborated or used the same underlying data (Madsen et al., 2020; Pilditch et al., 2020). These difficulties mean that people might believe that a consensus is not independent when it actually is, or that what looks like an independent consensus actually is not. For instance, most COVID-19 antivaccination views originated from the same few people (Center for Countering Digital Hate, 2021), and the majority of climate-change-denial blogs rely on the same few primary sources (Harvey et al., 2018).

Given these implications, it is vital to understand the extent to which people are actually sensitive to consensus independence when reasoning about real-world topics. Indeed, this question has been the focus of several recent experimental investigations. Much of this evidence suggests that people are insensitive to consensus independence except in specific contexts, such as when the claim is easily knowable (Aboody et al., 2022; Yousif et al., 2019), when independence is clearly emphasized (Connor Desai et al., 2022), or when the relationship between the source and the conclusion is clear (Alister et al., 2022)—but even then, the effect of source independence is usually very small.

However, the issue is still far from settled. First, because it is usually assumed that an independent consensus should be the more persuasive form of evidence, relatively little effort is usually made to ascertain the extent or nature of individual variation from this standard. Indeed, pure repetition of information, regardless of source independence, can also influence beliefs (e.g., Fazio et al., 2022; Pillai & Fazio, 2024). As discussed above, people might reasonably differ in how they use source independence to judge reliability or competence. This individual variation may look like a null or weak effect on the group level but reflects interesting and sophisticated reasoning at the individual level (Xie & Hayes, 2022).

A second limitation is that studies showing the “standard” consensus effect (i.e., stronger belief in claims that are endorsed by many people) have typically involved a limited number of claims and claim types. This issue is important because people might reason about consensus differently for different kinds of claims (Richardson & Keil, 2022). For instance, Yousif et al. (2019) found that when a claim was about a new tax policy and the sources were economists, people were more sensitive to dependence than when it was about an event at a local school and the sources were eyewitnesses. The authors concluded that people may reason differently about claims that are less knowable (like an economic prediction) than about those that have a clear ground truth (like eyewitness accounts). This hypothesis is consistent with work demonstrating that source expertise matters (Maddux & Rogers, 1980; Simmonds et al., 2024), probably in part because experts have more insight into the ground truth of a situation. However, claim knowability has never been systematically manipulated over a variety of claims, nor has this hypothesis been tested in a more standard consensus paradigm comparing contexts in which a consensus about a claim has or has not been reached. Hence, it remains unclear how much claim knowability matters or how robust this effect is.

The current study addresses these limitations and is the first to systematically investigate individual- and claim-level differences in consensus effects for a large number of realistic claims. To our knowledge, the current study involved the most stimuli by far in any experimental consensus-reasoning paradigm to date. Each of our participants read a total of 420 unique, realistic social-media posts, out of a pool of 1,080 total posts (to allow for appropriate randomization across conditions) for 60 unique claims (one claim per trial; see the Method section). Generating so many trials enabled us to implement reliable Bayesian analyses for each person. Including more claims also allowed us to develop and test a new taxonomy of claim types on the basis of their knowability, which meant we were able to systematically identify whether different kinds of claims elicited different consensus effects.

## Research Transparency Statement

### General disclosures

**Conflict of interest:** All authors declare no conflicts of interest. **Funding:** Manikya Alister was supported by an Australian Government Research Training Program Scholarship. **Artificial intelligence:** Artificial intelligence (ChatGPT) was used to generate some of the fake social-media posts used in the experiment. (More details are provided in the Procedure section.) However, the originally generated posts were later refined manually to ensure realism and variety. No other artificial-intelligence-assisted technologies were used in this research or the creation of this article. **Ethics:** Our study was approved by the University of Adelaide Ethics Committee (Approval No. 20/78).

### Study disclosures

**Preregistration:** Our research aims, experimental design, and most of our analyses were preregistered on January 8, 2024. Primary data collection began on January 10. All preregistered analyses were performed as described with no deviations, and analyses that were not preregistered are clearly specified when mentioned in the main manuscript. Our preregistration can be found here: aspredicted.org/7pdm-vgpx.pdf. As noted in our preregistration, we collected pilot data from 5 participants to ensure that the experiment was running correctly, and our estimates about how long it would take were correct. We did not make any changes to the experiment after the pilot experiment, so these participants were included in the final sample. **Materials:** All of the experimental material (social-media posts, university/media institutions, user names, and photos) are publicly available at osf.io/mtuyv/. **Data:** All anonymized primary and clean data (including cleaning scripts) are available at osf.io/mtuyv/. **Analysis scripts:** All analysis scripts necessary to reproduce the results are available at osf.io/mtuyv/. **Computational reproducibility:** The computational reproducibility of the results has been independently confirmed by the journal’s STAR team.

### Method

Our study was approved by the University of Adelaide Ethics Committee (Approval No. 20/78).

### Participants

Participants (*N* = 160) were recruited from Prolific Academic. Of those participants, 116 completed Session 1 and were paid £5.25 per session for up to two 35-min sessions. We removed 38 participants on the basis of our preregistered exclusion criteria; we removed an additional 25 participants who had less than 90% accuracy in comprehension checks in Session 1 (see the Procedure section) and were not invited to complete Session 2 (2 pilot participants did both sessions despite having low accuracy in the first session, but they were still removed from analyses). Of the remaining participants who were invited to complete Session 2, a further 2 participants were removed because of low accuracy, and 11 were removed because they failed to complete Session 2, leaving a total of 78 participants. Ages ranged from 18 to 72 years old (*M* = 36 years) with 42 participants identifying as female, 34 as male, and 2 as other. All were native English speakers who used English as their primary language.

### Procedure

**Fig. 1.**
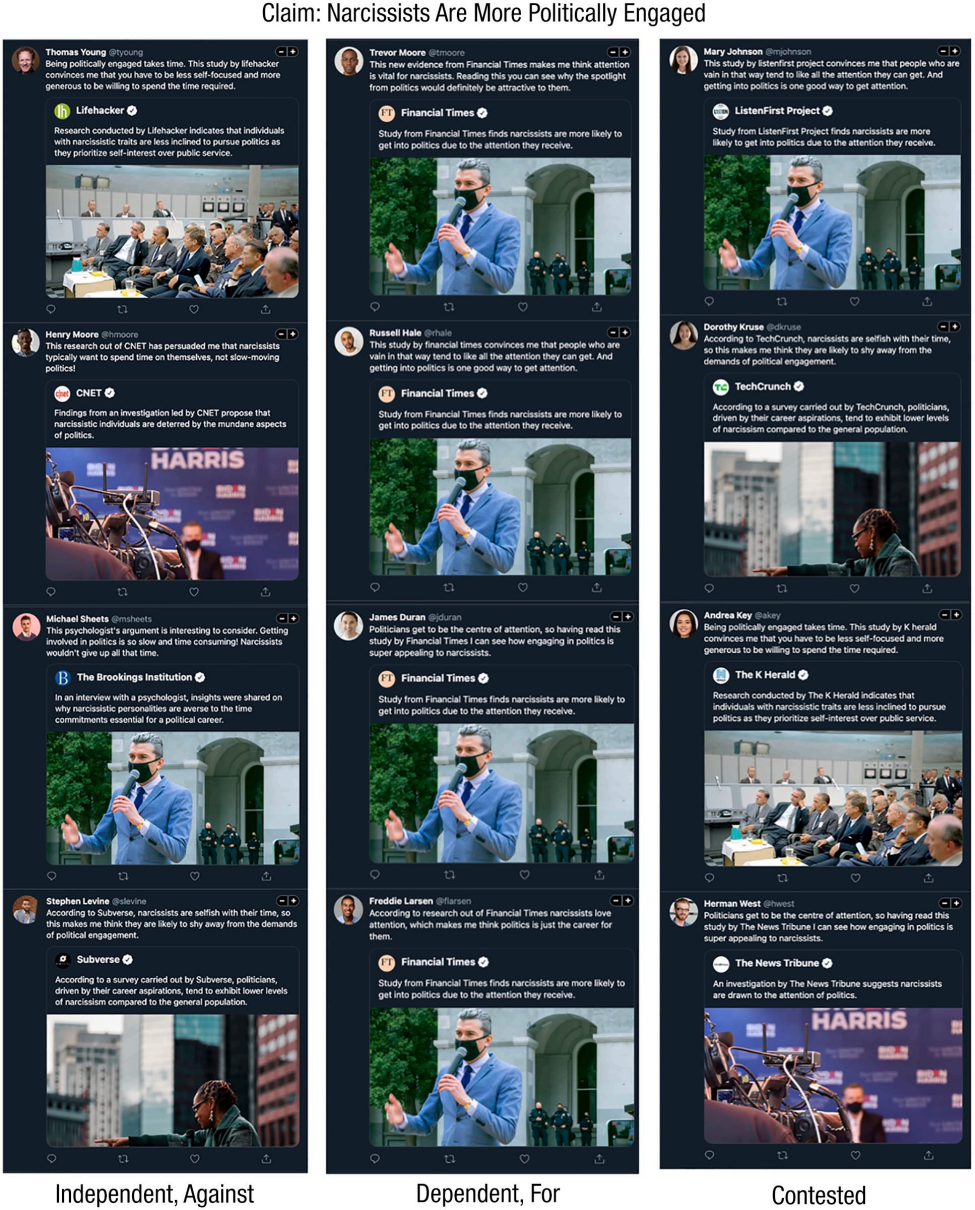
Experiment stimuli with sample posts from each consensus condition. The left panel is from the independent-consensus condition, in which each person re-posted a different source (here, arguing against the claim, or “against”). Those in the middle are from the dependent-consensus condition, in which each person re-posted the same source (here, arguing for the claim, or “for”). The right column shows the contested condition, in which an equal number of people supported or opposed the claim. The sources, names, photos, text, dependence, and support direction were randomized for each person and claim. In this example the sources are news organizations, but for some claims they were universities.

After providing consent and passing a short quiz regarding the task instructions, each participant saw 60 trials over the course of two sessions on two separate days (30 trials per day). Our choice in the number of trials was informed by a simulation-based power analysis (see Supplemental Material 2 in the Supplemental Material available online). Each trial began with participants viewing a claim (e.g., “Narcissists are more politically engaged”). The claim was accompanied by a photo and a brief, neutral social-media post pertaining to the claim (e.g., “I’m a modest person, many people say I’m incredibly modest, so I found this interesting to consider . . .”) to help set the scene for the participants and facilitate engagement with the claim. After reading the claim, they were asked to indicate “To what extent do you agree with the claim?” using a slider ranging from 0 to 100. They then viewed four social-media posts by four distinct users who were each sharing another post from a primary source (see Fig. 1). To ensure that people were engaging with the task properly, we required participants to indicate whether each user was arguing for or against the claim. The 27 participants who were less than 90% accurate at this task were removed from the analysis.

As shown in Figure 1, each post took the form of a re-post and included the primary source being re-posted, the primary data referred to by the source, and the user’s own words explaining how the primary source was persuasive. Because the four users were always distinct people with unique profile photos and names, this made it clear that all users had read the source and that it had influenced their opinions about the claim; this was shown to be important in previous work (Alister et al., 2022).

After confirming that they had read each post (by indicating whether the post endorsed or opposed the claim), participants were once again asked to indicate how much they agreed with the claim (using the same 0–100 slider as before, but initialized to indicate their initial rating). The difference between participants’ rating before and after seeing the claims represented their degree of belief revision after reading the posts. After completing all 60 trials of the experiment, they were given a multiple-choice question about which strategy they used to evaluate the claims (see the Results section for full details of the question and the response options).

### Consensus conditions

Our primary manipulation (within participants) pertained to the presence or absence of a consensus within the four social-media posts as well as—if there was a consensus—whether or not it was formed independently. Specifically, trials were either consensus trials, in which everyone reached the same opinion, or contested trials, where an equal number of posts supported and opposed the claim (right column of Fig. 1). There were two kinds of consensus trials: independent consensus and dependent consensus (20 trials each per person). In independent trials, each of the four social-media posts cited different primary sources (see the left column of Fig. 1). In dependent trials, each of the four social-media posts cited the same primary source (see the middle column of Fig. 1). The stance of the consensus was randomized, with the constraint that there was always an equal number of supporting (for) and opposing (against) trials for each claim type and consensus condition.

The 20 contested trials were included for two reasons. First, they allowed us to investigate individual differences in the strength of standard consensus effects (i.e., how many people showed a larger belief shift in full-consensus vs. no-consensus trials). Second, it served to reduce demand effects and ensure that participants read all of the posts; otherwise, they could pass the manipulation check without reading them once they realized that all posts already agreed with the first one.

Regardless of condition, all four posters and sources gave essentially the same reason in different words (e.g., those arguing “for” on the narcissism claim all pointed out that narcissists were more likely to get into politics because of the attention they receive, and those arguing “against” all pointed out that the amount of time required to engage in politics is unappealing for narcissists; see Fig. 1). The order of claims and posts, the assignment of claims to condition, and the assignment of avatars and names were randomized across all participants and claims. The primary source was always either a news organization or a university, and the primary data was always some kind of study or investigation carried out specifically by that organization (made clear through the wording of the post). For example, the primary data could be a study by the University of Springfield, and the primary source would be the official account of the University of Springfield.

In the dependent condition, each of the four posts (by four different users) re-posted the same article by the same source (hence the same primary data). In the independent and contested conditions, the source of the post that was re-posted and the data that the source referred to were both distinct for each of the four posts: Person A cited Source X, Person B cited Source Y, and so forth. Thus, each was re-posting an independent source and referring to independent primary data. There were always three primary sources and one expert testimony, which was included to add some variety and reduce demand effects. We tried to maintain a balance between how many trials used each source type, but in some cases it only made sense to have a particular source type (e.g., a university would not conduct a study about whether a mayor ran into a burning building). In total, 27 of the claims had universities as their primary sources, and 33 had news organizations.

The news companies were real media companies chosen via the website AllSides,^1^ which allows people to rate the bias of different news companies. We chose news companies that were mid-range in popularity and deemed “centrist” by the raters. The universities were a sample of real universities ranked between 100 and 200 by the QS World University Rankings. These universities were chosen because they would be recognizable enough to be believable but not so elite that their reputation would substantially influence people’s beliefs. Whether the sources were news companies or institutions was deliberately chosen for each trial on the basis of the appropriateness to the claim, but the actual company or institution included in each trial was randomized for each participant and was always unique. The companies or institutions always had a profile photo, full name, and a “verified” tick to signal authenticity. All content was fictitious. The content that was not part of Alister et al. (2022) was first generated using ChatGPT-3.5 Turbo-1106 and then refined manually to ensure realism and variety.

### Claim-type conditions

In addition to varying the nature of the consensus across conditions within participants, we also varied the nature of the claims themselves. Given that previous research has suggested that knowable claims are more likely to induce consensus independence effects (Yousif et al., 2019), we selected 30 *knowable* claims and 30 *unknowable* ones. Each category was further subdivided on the basis of the way in which it was knowable or not. For instance, *knowable eyewitness* claims include something that somebody could have seen (e.g., a mayor saving a child from a burning building). A *knowable fact* is something that, although verifiable in principle, you would need to be an expert to confirm (e.g., a new species of jellyfish being discovered). An *unknowable expert* claim is one that does not have a known ground truth at the moment but that can be best evaluated through special expertise (e.g., economic forecasting). Last, *unknowable preference* claims do not have a ground truth, and expertise is less likely to be important (e.g., whether flying is a better superpower than invisibility). Thus, all of the 60 total trials per participant were composed of 15 trials of each of the four claim types. Given previous literature (Yousif et al., 2019), we expected that knowable claims would result in larger consensus effects compared with unknowable claims. The range and strength of prior beliefs endorsed by participants varied considerably across claims and within claim types. (See Supplemental Material 6 for the full set of claims and the distribution of people’s initial belief ratings for them.)

## Results

### Aggregate behavior

We first examined the extent to which belief revision was affected by the knowability of a claim and the type of consensus. To account for the fact that “against” trials (in which the consensus argued against the claim) would shift beliefs in the opposite direction, we reversed the belief scores for against trials for the purpose of analysis so that a positive shift always meant an update in the direction of the consensus. Effect sizes were equivalent for for and against trials across conditions (see Supplemental Material 7.1).

**Fig. 2.**
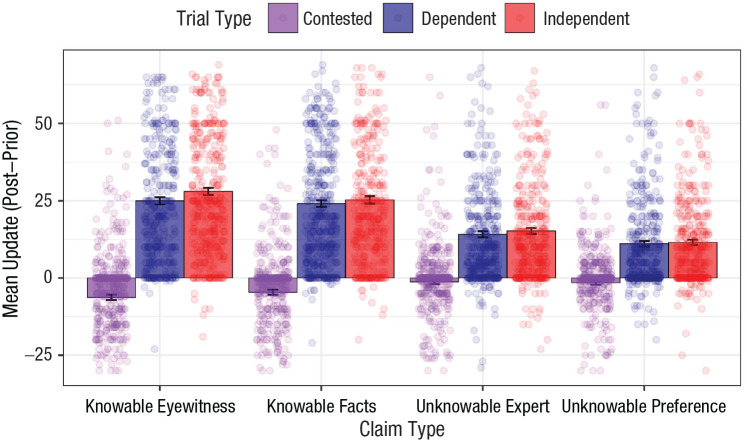
Belief update as a function of trial type and claim type. Positive belief update indicates belief change in the direction of the consensus for the full consensus trials. Independent trials (red) showed four users who shared the same opinion but cited different primary sources. Dependent trials (blue) involved four users who shared the same opinion and cited the same primary source; contested trials (purple) involved four users, half agreeing with the claim and half disagreeing, with each user citing different sources. The axes have been slightly constrained to better show the mean differences, so some individual data points are not visible.

As Figure 2 shows, the largest belief updates occurred in the full consensus trials (independent and dependent, in which all four posts took the same stance toward the claim). People were much more persuaded by these trials than by contested ones in which half of the posts argued in one direction and half in the other. In the contested trials, people actually tended to believe the claim less after seeing the posts, although the degree of belief revision was very small. The figure also suggests the presence of a small but consistent difference between independent and dependent consensus trials, with participants on average more convinced when sources were independent.

To quantitatively assess the persuasiveness of different kinds of consensus as a function of the type of claim, we compared four nested Bayesian generalized linear models using the *brms* package (Version 2.20.4; Bürkner, 2018) in R (Version 4.2.2), using default priors: a weakly informed student’s *t* distribution for the intercept, and a uniform (flat) prior for the coefficients (slopes). Inspection of convergence metrics, such as $\hat{R}$ , suggested that using flat priors did not cause any problems with convergence in the sampling process.

The outcome variable was the rating after reading the four posts, and people’s initial beliefs (before seeing the posts) were included as predictors in all models (e.g., Table 1). To assess the relative performance of each model, we compared them using the leave-one-out cross-validation criterion (LOOIC; Vehtari et al., 2017). Our focus was on two kinds of consensus effects: standard consensus (independent trials vs. contested trials) and consensus independence (independent trials vs. dependent trials). We therefore ran each of the models on the relevant subset of data, as described in the next section. Although we did not conduct a simulated power estimation for the analyses of the aggregate behavior, as we did for the individual-level analyses (see Supplemental Material 2), we expected this sample size to be sufficient for calculating group-level effects given the number of trials per person, because previous studies using the same design had been able to detect effects similar size with around one quarter of the number of total observations in our study (e.g., Alister et al., 2022).

**Table 1.** Unstandardized Coefficients for the Best Model Comparing Independent to Contested

Coefficient	Estimate	89% Credible Interval	
		Lower	Upper
Intercept	9.54	7.82	11.26
Initial Belief	0.72	0.70	0.73
Consensus_*Ind*_	32.90	31.12	34.68
Claim Type_*KnowFact*_	1.91	0.03	3.76
Claim Type_*UnknowExpert*_	6.40	4.59	8.21
Claim Type_*UnknowPref*_	4.21	2.33	6.02
Consensus*_Ind_ ×* Claim Type_*KnowFact*_	−4.65	−7.20	−2.06
Consensus*_Ind_ ×* Claim Type_*UnknowExpert*_	−18.78	−21.36	−16.17
Consensus*_Ind_ ×* Claim Type_*UnknowPref*_	−21.49	−24.13	−18.80

Note: Because initial belief is a continuous variable on the same scale as the outcome measure, it is interpreted this way: Every unit increase in people’s initial beliefs was associated with a .72 increase in their beliefs after seeing the posts (suggesting that participants’ initial beliefs accounted for quite a lot). The coefficients of the categorical variables are interpreted with respect to their reference variables, which is the level of the variable not contained in the table. Further, because of the default dummy coding of the interaction model, the main effects are interpreted at the interacting variable’s reference level, rather than aggregating across the other variable. Consequently, 32.9 for Consensus_*Ind*_ means that participants rated independent trials as 32.9 units more convincing than contested trials when the claim type was a knowable eyewitness. For the same reason, the claim-type coefficients reported here correspond to their values when the consensus trial is contested, which is why the estimates suggest that knowable-expert trials were the least convincing, even though they were the most convincing when we aggregated across all independence conditions (see the last page of Supplemental Material 3 for full post hoc comparisons). Following Kruschke (2014), we show 89% credible intervals in all analyses (see simulated power estimation in Supplemental Material 2 for further justification). Ind = independent; KnowFact = knowable fact; UnknowExpert = unknowable expert; UnknowPref = unknowable preference.

As mentioned earlier, we chose a Bayesian statistical approach, both here and in the individual-level analyses presented later. We chose this instead of a frequentist approach for several reasons. First, Bayesian models estimate parameters by calculating the posterior probability distribution for each parameter, which gives the probability of different parameter values given the data and our prior expectations (for an overview of Bayesian analysis, see McElreath, 2016). This contrasts with the frequentist approach, which focuses on testing hypotheses and provides *p* values, or the probability of observing data as extreme as the observed data under the null hypothesis. Frequentist methods, such as null hypothesis significance testing, only allow us to reject or fail to reject the null hypothesis, often with the sole conclusion of whether a parameter is different from zero (e.g., *p* < .05).

In contrast, Bayesian methods allow us to quantify the uncertainty surrounding the entire range of possible parameter values, as opposed to merely indicating whether a parameter is significantly different from zero. Bayesian analyses yield credible intervals—the range of parameter values within which the true value lies with a specific probability, given the data and the model. For example, the 89% credible intervals we report in Tables 1 and 2 and Figure 4 mean that there is an 89% probability that the true parameter lies within this range, given the observed data and prior assumptions (we provide an explanation for why we use 89% in Supplemental Material 2).

**Table 2.** Unstandardized Coefficients for the Best Model Comparing Dependent to Independent

Coefficient	Estimate	89% credible interval	
		Lower	Upper
Intercept	42.896	40.789	44.977
Initial Belief	0.666	0.648	0.684
Consensus_*Ind*_	1.678	0.725	2.620
Claim Type_*KnowFact*_	−2.706	−4.055	−1.329
Claim Type_*UnknowExpert*_	−12.420	−13.816	−11.054
Claim Type_*UnknowPref*_	−15.898	−17.271	−14.512

Note: Ind = independent; KnowFact = knowable fact; UnknowExpert = unknowable expert; UnknowPref = unknowable preference.

This interpretation is more intuitive than the frequentist 95% confidence interval, which is often misunderstood. A confidence interval reflects the range within which the parameter would fall if one could repeatedly sample from the population and calculate estimates in each sample. It is important to note that a confidence interval does not provide direct probabilistic information about the parameter given the data at hand, which is what Bayesian credible intervals do give us.

### Comparing beliefs in full consensus versus no consensus

We first asked whether people changed their belief more when the four posts agreed (a full consensus) than when half argued in one direction and half in the other (no consensus). In order to quantitatively test this, we compared the independent condition trials to the contested condition trials that were directly comparable in other ways: In both conditions, the four posts had distinct, independent sources. (Dependent trials differed from contested trials in having a single repeated source and were thus excluded from this comparison.)

Results of the full model comparison are in Table 3. The best model (Model 4), whose coefficients are reported in Table 1, had credible main effects of both consensus type (independent vs contested) as well as an interaction with claim type. The main effect of consensus suggests that people were more convinced by independent trials compared with contested trials. The main effect of claim type suggests that people were most convinced by knowable eyewitness claims, followed by knowable facts, then unknowable expert, and lastly unknowable preference. Follow-up comparisons, reported in Supplemental Material 3, revealed credible differences between each claim type. Model 1 only considered participants’ initial beliefs about the claim. Model 2 also considered whether there was a consensus or not, and Model 3 added claim type. Model 4 also considered the interaction between the presence of a consensus and claim type and was favored by leave-one-out cross-validation criterion (LOOIC). All models also contained a random intercept term for each participant (Brown, 2021).

**Table 3.** Model Comparison Predicting Belief in the Claim After Independent Versus Contested Trials

Model	LOOIC	*SE*	Rank
Model 1: Initial Belief	27693	100	4
Model 2: Initial Belief + Consensus	26633	116	3
Model 3: Initial Belief + Consensus + Claim Type	26565	117	2
Model 4: Initial Belief + Consensus × Claim Type	**26335**	121	1

Note: LOOIC = leave-one-out cross-validation criterion. The lowest LOOIC (indicating the best model performance) is in bold.

The model also suggested an interaction between the nature of the consensus and the type of claim: The difference in belief between a full consensus and no consensus was larger when the claims were more knowable. Interestingly, looking at Figure 2, this interaction reflects not only the fact that for knowable claims people were more convinced by a consensus, but also that they were more likely to revise their beliefs against knowable claims on contested trials. One might imagine that this reflects initial differences in certainty between knowable and unknowable claims. However, this does not appear to be the case: When we added prior certainty to the models (operationalized as the distance of initial beliefs to the initial belief scale midpoint (as in Orticio et al., 2022), the main effects and interactions remained (see Supplemental Material 4.3). Instead, it is possible that the presence (or absence) of a consensus is more informative for knowable claims. If a claim is unknowable in principle, it does not mean much whether people agree or disagree. Conversely, full consensus for a knowable claim might be reasonable evidence that the claim is true, whereas disagreement on knowable claims is a good indication that it might not be.

### Comparing beliefs in dependent versus independent consensus

Our second question concerned whether people reasoned differently for independent and dependent trials. As shown in Table 4, the full-model comparison favored Model 3, indicating that the independence of the consensus and the type of claim were both important to belief revision (with no interaction): On average, people tended to be more convinced by an independent consensus than a dependent one. The main effect of claim type reflected a larger effect for knowable than unknowable claims (Fig. 2). Although the main effect of independence was credibly greater than zero in the winning model, it was quite small relative to the other consensus comparison (as evident in Fig. 2 as well as in the coefficients reported on page 2 of Supplemental Material 3).

**Table 4.** Model Comparison Predicting Belief in the Claim After Independent Versus Dependent Trials

Model	LOOIC	*SE*	Rank
Model 1: Initial Belief	26952	103	4
Model 2: Initial Belief + Independence	26946	103	3
Model 3: Initial Belief + Independence + Claim Type	**26493**	111	1
Model 4: Initial Belief + Independence × Claim Type	26496	111	2

Note: The models were similar to those shown in Table 3, but here we compare the two independence conditions. Model 3 was favored by leave-one-out cross-validation criterion (LOOIC). The lowest LOOIC (indicating the best model performance) is in bold.

A follow-up analysis indicated that the group-level results were qualitatively unchanged even on the first trial, suggesting that they did not arise because of demand effects or because participants were becoming sensitized to the consensus manipulations over the course of the experiment (the full analysis is reported in Supplemental Material 7.2).

### Individual differences

**Fig. 3.**
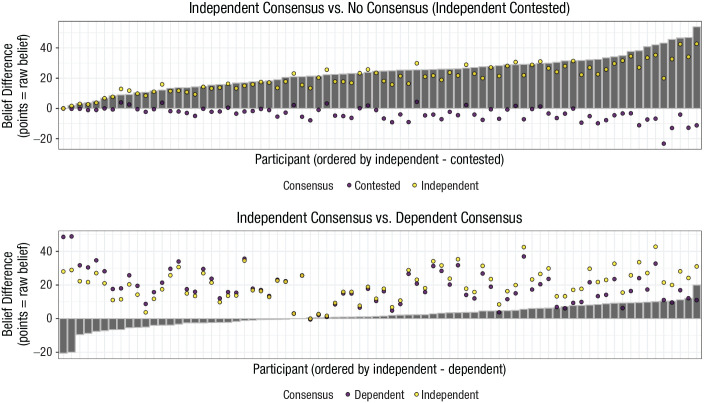
Belief updates for each individual. Bars represent the difference in beliefs across the comparison conditions: independent – contested (top), independent – dependent (bottom). Dots represent the raw average change in belief toward the direction of each respective consensus condition (independent consensus, dependent consensus, or contested). The plot shows that almost everyone shifted their beliefs more on average for independent trials compared with contested trials (top), but there was substantial variability in how persuaded people were by independent trials compared with dependent trials (bottom).

A key aim of this experiment was to explore whether and how individuals vary in how they reason about consensus. We therefore looked at individual behavior on the same questions as before: To what extent do people change their beliefs more when there is a full consensus versus no consensus, or an independent versus dependent one? As Figure 3 shows, the vast majority of people were sensitive to consensus, changing their belief more when all posts agreed; however, people varied substantially in how they reasoned about independence.

A detailed note on how to interpret the coefficients is found in Table 1. The only difference is that because the best-performing model did not contain an interaction, the main effects of the categorical variables are collapsed across the levels of the other variable.

We used Bayesian linear models to quantitatively classify the different kinds of participants as one of four possible logical types. First, some people might be generally insensitive to consensus: they reason similarly regardless of whether all four people agree or whether there are two on each side. Second, some people might be sensitive to the presence of a consensus but insensitive to source independence. Third, some people might be more convinced by a consensus when the sources are independent. And finally, some might be more convinced when the sources are dependent. Each participant was fitted to a model in which the outcome variable was that person’s belief after seeing the posts. We then compared the two models: a baseline null model, in which the only predictor was that person’s prior belief in the claim, compared with an alternative model that also included a predictor corresponding to the effect in question.

**Fig. 4.**
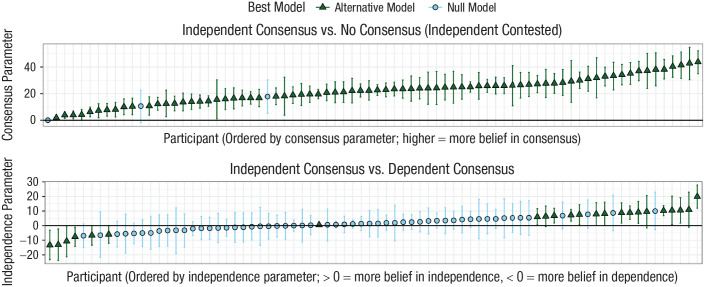
Individual-level modeling results. At the top, we show for each person the estimated persuasiveness of an independent consensus relative to a contested consensus (*x*-axis). The *y*-axis indicates a parameter reflecting the extent to which a participant was more persuaded by a full consensus. The parameter is shown by the point and its 89% credible interval by the lines. Blue circles indicate those participants whose behavior was best captured by a null model containing only their initial beliefs. Green triangles represent participants who were more persuaded by consensus. At the bottom, we show for each person the estimated persuasiveness of an independent consensus relative to a dependent consensus, with green triangles representing participants who were sensitive to independence in either direction.

The top panel of Figure 4 explores sensitivity to consensus (i.e., whether people changed their beliefs more with a full consensus than with none). It shows, for each person, whether they were best fit by the null model (blue circle) or by the alternative (green triangle) that included a predictor corresponding to consensus (contested vs independent). All but three participants (96%) were best described by the alternative model, suggesting that the vast majority of people were more convinced by four people agreeing than by two people on each side. The median individual difference in belief change between the full consensus (independent) trials and the contested trials was 23 points in favor of the independent trials. There was substantial individual variation, however, ranging from close to zero change to over 40 points of shift.

The bottom panel of Figure 4 explores sensitivity to independence. It shows, for each person, whether they were best fit by the null model or an alternative model that included a predictor corresponding to independence (dependent vs. independent). The majority of people were best fitted by the null model, but 22% were more persuaded on independent trials (positive on the *y*-axis) and 8% were more persuaded on dependent trials (negative on the *y*-axis). Although the majority of participants showed no sensitivity to independence, these results support the idea that there are notable individual differences. For example, at the group level, median belief change was only 2 points higher on independent trials, but for participants best fit by the alternative model it was 9 points. In addition, the median estimate of belief change for those who were more convinced by a dependent consensus was just as strong (also 9 points).

In Supplemental Material 1, we present various exploratory analyses (not preregistered), investigating whether these individual differences are associated with any of the demographic variables we collected (age, education, politics, and social-media use). However, our analyses did not reveal any statistical evidence that would allow us to reliably conclude that any of these variables were driving individual differences. However, it is important to emphasize that our study was not designed to test these relationships, so failure to find any credible relationships in our analyses does not mean they do not exist. However, these analyses may be useful for motivating future researchers interested in more rigorously testing what underlies individual differences in the persuasiveness of different kinds of consensus.

One consideration is that because claims were randomized for each person, different people saw different claims in each consensus condition. Therefore, it is possible that the individual variation we observed could be a reflection of the fact that some participants were differentially exposed to the claims likely to produce independence effects. If this is indeed an issue, one indicator would be individual-level results that were driven by a small number of trials, or claims in which participants showed particularly large belief changes. In Supplemental Material 5 we also report a number of follow-up analyses (not preregistered) which suggested that this individual variation was not due to a few outlier claims and instead reflected consistent preferences for a particular type of consensus.

As an exploratory (not preregistered) analysis of consistency between the two sessions, we also ran the model on each session separately to see whether people who were judged as being sensitive to independence overall exhibited similar behavior in both sessions. Although the estimates were substantially noisier because there were fewer trials, there was a strong positive correlation, *r* = .57, *t*(21) = 3.07, *p* = .006, between estimates in the two sessions for participants who were best fitted by the alternative model overall. In other words, people who changed their beliefs more in the direction of independent trials in the first session also did so in the second session, and the reverse was true for people who changed more in the direction of dependent trials. As one might expect, participants who were classified in neither group did not appear to have any consistent preference across sessions.

### Exploratory analysis: How often did people change their minds?

**Fig. 5.**
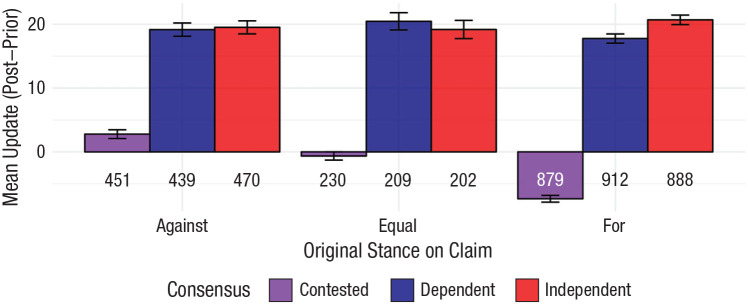
Belief updates as a function of whether the participant originally believed the claim (for), disbelieved (against), or neither (equal). Numbers represent the number of observations (trials) in each cell. Error bars represent standard errors.

We have shown that people usually updated their beliefs in line with the consensus but that the size of this change varied depending on how knowable the claim was. What remains unclear is (a) how belief changes varied depending on whether people initially agreed with the claim or not and (b) how often people actually changed their minds qualitatively by switching from disbelieving to believing, or vice versa, as opposed to just reducing their certainty in their original belief. Figure 5 shows how much beliefs changed, on average, on the basis of whether people originally believed the claim (for; belief > 50), disbelieved (against; belief < 50), or neither (equal; belief = 50). Interestingly, there only appeared to be a reasonably clear group-level difference between independent and dependent trials when participants were initially in favor of the claim. In the contested trials, people were more likely to change their beliefs in favor of the claim if they were originally against it, and they were more likely to revise their beliefs against the claim if they were originally in favor. The directionality of these differences can probably be partially explained by ceiling effects (people had more room to move on the scale in the opposite direction to what they already believed). However, ceiling effects do not explain why the belief revision was more pronounced when participants were originally for the claim than when they were initially against it.^2^

**Fig. 6.**
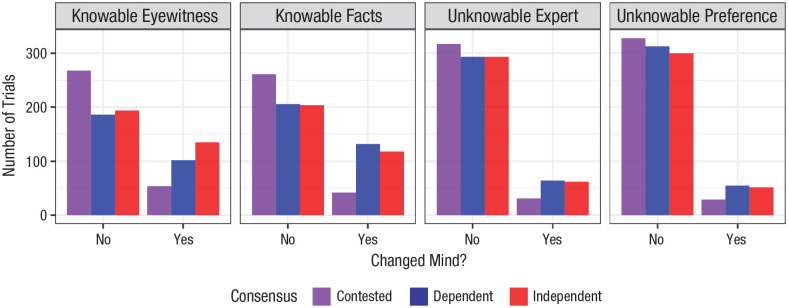
The number of participants who changed their minds (switching from believing to disbelieving, or disbelieving to believing) after viewing the posts. Trials in which participants had an initial rating of 50 (i.e., they neither believed nor disbelieved) were removed from this analysis (see Fig. 5 for the number of trials in which participants had no prior preference).

Another way to explore the same issue is shown in Figure 6, where we illustrate the number of trials in which participants qualitatively changed their minds by switching either from believing to disbelieving or from disbelieving to believing. (Participants who had a prior rating of 50, indicating no preference, were removed from this analysis.) Participants changed their minds on 22% of trials, with 82% of those changes of mind occurring in the consensus conditions. This result shows that not only did some people update their beliefs in line with the consensus, but they actually qualitatively changed their minds. Further, the extent to which people changed their minds corresponded with how knowable the claim was: People were more likely to change their minds for knowable claims (7% for knowable eyewitness and 7% for knowable fact) compared with unknowable claims (4% for unknowable fact and 3% for unknowable preference).

## Discussion

We performed a large-scale assessment of consensus persuasiveness over many different individuals and claim types. Using a mock social-media paradigm in which each participant assessed the veracity of 60 realistic claims, participants updated their beliefs in light of either (a) an independent consensus (everyone agreeing and citing different sources), (b) a dependent consensus (everyone agreeing but citing the same sources), or (c) no consensus (two in favor, two opposing, all citing different sources). These three trial types allowed us to look at two kinds of consensus effects, which we discuss below.

### Full consensus versus no consensus

We were able to replicate previous work demonstrating large group-level belief changes in the direction of claims that are supported by a consensus relative to no consensus (e.g., Ransom et al., 2021). In addition, unlike previous research, we were able to quantify this at the individual level: Nearly everybody updated their beliefs in line with the consensus, with substantial variation in how much.

We also found that this effect was stronger for claims that are more knowable—that is, claims that are more likely to have a ground truth. Although this possibility has been suggested to underlie independence effects (Yousif et al., 2019), we are the first to (a) develop an explicit taxonomy that distinguishes between different kinds of knowability and (b) show that this taxonomy can reliably predict how persuasive a standard consensus is. This finding makes sense; if it is impossible for anybody to know the truth of a claim, an aggregate of opinions might not be very convincing. Conversely, it might be more sensible to aggregate opinions when there is a knowable ground truth: In such cases, consensus estimates are consistently more reliable than individuals (e.g., Mannes et al., 2014).

The fact that knowability is relevant has important implications. It suggests that if bad actors want to reduce people’s belief in a claim that has a consensus among experts, they do not necessarily need to reduce the perception that a consensus exists; they might merely need to create doubt that the claim is knowable at all. Indeed, this kind of rhetoric is already employed by popular climate deniers with wide-reaching platforms (e.g., Jordan Peterson), who have claimed that the climate is too complicated to be modeled accurately (e.g., Readfearn, 2022).

### Independent versus dependent consensus

Consistent with a number of recent studies, we found a small effect of consensus independence. At the group level, people tended to be more convinced when everybody cited different sources rather than the same ones (Connor Desai et al., 2022; Simmonds et al., 2023; Yousif et al., 2019). Interestingly, this group-level effect was not influenced by the claim type, contrary to the hypothesis posited by Yousif et al. (2019).

At the individual level, most participants in our study were similarly persuaded by an independent consensus rather than a dependent consensus, but the minority that were more persuaded by independence updated their beliefs more than our group-level effects would suggest. Further, a small subset of participants were more convinced by a dependent consensus (to an equivalent degree as those who preferred an independent consensus). These individual differences highlight how small or null group-level effects can be misleading (such as those found in our own study and previous studies, e.g., Alister et al., 2022; Connor Desai et al., 2022; Sulik et al., 2020; Yousif et al., 2019), because they hide the heterogeneity in individuals’ reliance on a given factor.

The majority who did not prefer an independent consensus is particularly interesting given that dominant normative computational theories of reasoning outline that people should be more convinced by independent evidence (Couch, 2022; Madsen et al., 2020; Whalen et al., 2018), or simply assume independence without considering contexts where this may not be the case (e.g., Oktar et al., 2024). Our findings have at least two possible interpretations with respect to these theories. One interpretation of our results, which is how most research has described deviations from normative models in these contexts, is that it shows how people are often irrational when reasoning from a consensus and do not appropriately weight the informational advantages gained by independence. Our individual-level approach, however, allows for a second interpretation, which is that people have different underlying (but reasonable) assumptions for what independence versus dependence means. For example, observing multiple people endorsing the same primary source could be a cue to that source’s reliability; if that is the case, and if source reliability is uncertain enough, a dependence consensus might actually be more persuasive. Variation in the extent to which people make this (or other) assumptions may contribute to the individual variation that we observed. This has important implications for existing theories of consensus reasoning, because it suggests that there are reasonable generative assumptions people are making that existing theories do not yet consider.

One consideration is that participants who could not accurately identify the stance of a post were removed. This could have inflated the standard consensus effect relative to the independence effect if it made people focus more on consensus than they otherwise would have. However, we believe this is unlikely, as our effect size aligns with previous studies that lacked this manipulation check (Alister et al., 2022; Ransom et al., 2021). An open question is whether similarly directing attention to independence would increase sensitivity to it. Additionally, it is also important to caveat that our sample was limited to U.S. participants recruited from Prolific, so our findings may not generalize outside of this sample.

In summary, learning how people are persuaded by different kinds of social consensus is crucial for understanding how information circulating online will affect public opinion (Lewandowsky et al., 2019). The current work used empirical and analytic methods that allowed us to examine the impact of different types of consensus on belief at both the group and individual levels. Our finding that individuals vary considerably in their sensitivity to source independence highlights how group-level analyses (which are the norm in this area) can obscure important individual heterogeneity that might be important for illuminating how different people reason about consensus information in the real world.

## Supplemental Material

sj-docx-1-pss-10.1177_09567976251344549 – Supplemental material for How Convincing Is a Crowd? Quantifying the Persuasiveness of a Consensus for Different Individuals and Types of Claims Supplemental material, sj-docx-1-pss-10.1177_09567976251344549 for How Convincing Is a Crowd? Quantifying the Persuasiveness of a Consensus for Different Individuals and Types of Claims by Manikya Alister, Keith Ransom, Saoirse Connor Desai, Ee Von Soh, Brett Hayes and Andrew Perfors in Psychological Science

## Transparency

*Action Editor:* Clayton R. Critcher

*Editor:* Simine Vazire

*Author Contributions*

**Manikya Alister:** Conceptualization; Data curation; Formal analysis; Funding acquisition; Investigation; Methodology; Project administration; Software; Visualization; Writing – original draft; Writing – review & editing.

**Keith Ransom:** Conceptualization; Methodology; Software; Supervision; Writing – review & editing.

**Saoirse Connor Desai:** Conceptualization; Methodology; Writing – review & editing.

**Ee Von Soh:** Methodology.

**Brett Hayes:** Writing – review & editing.

**Andrew Perfors:** Conceptualization; Formal analysis; Funding acquisition; Methodology; Supervision; Writing – review & editing.

**Artificial intelligence:** Artificial intelligence (ChatGPT) was used to generate some of the fake social-media posts used in the experiment. (More details are provided in the Procedure section.) However, the originally generated posts were later refined manually to ensure realism and variety. No other artificial-intelligence-assisted technologies were used in this research or the creation of this article.

**Ethics:** This study was approved by the University of Adelaide Ethics Committee (Approval No. 20/78).

**Open Practices:** To ensure long-term preservation, all Open Science Framework (OSF) files were registered at osf.io/f92ja. Study disclosures. Preregistration: Our research aims, experimental design, and most of our analyses were preregistered on January 8, 2024. Primary data collection began on January 10. All preregistered analyses were performed as described with no deviations, and analyses that were not preregistered are clearly specified when mentioned in the main manuscript. Our preregistration can be found here: aspredicted.org/7pdm-vgpx.pdf. As noted in our preregistration, we collected pilot data from 5 participants to ensure that the experiment was running correctly, and our estimates about how long it would take were correct. We did not make any changes to the experiment after the pilot experiment, so these participants were included in the final sample. Materials: All of the experimental material (social-media posts, university/media institutions, user names, and photos) are publicly available at osf.io/mtuyv/. Data: All anonymized primary and clean data (including cleaning scripts) are available at osf.io/mtuyv/. Analysis scripts: All analysis scripts necessary to reproduce the results are available at osf.io/mtuyv/. Computational reproducibility: The computational reproducibility of the results has been independently confirmed by the journal’s STAR team.

## Notes

1.
allsides.com/unbiased-balanced-news

2. None of the analyses that appear in this section were preregistered.
