# Supplementary material for: How Convincing Is a Crowd? Quantifying the Persuasiveness of a Consensus for Different Individuals and Types of Claims
Source: Psychol Sci. 2025 Jun 11;36(7):483–98. doi: 10.1177/09567976251344549 (PMC13428892; doi:10.1177/09567976251344549)
Supplement: sj-docx-1-pss-10.1177_09567976251344549 – Supplemental material for How Convincing Is a Crowd? Quantifying the Persuasiveness of a Consensus for Different Individuals and Types of Claims [file sj-docx-1-pss-10.1177_09567976251344549.docx]

Supplemental files can be found at [OSF | Individual and Topic Level Differences in Sensitivity to Consensus Effects Online](https://osf.io/mtuyv/)
